# Task-irrelevant abrupt onsets differentially impact value-related orienting and maintenance

**DOI:** 10.3758/s13414-025-03078-7

**Published:** 2025-05-13

**Authors:** Carly Chak, Emily Machniak, Barry Giesbrecht

**Affiliations:** Department of Psychological and Brain Sciences, Institute for Collaborative Biotechnologies, University of California, Santa Barbara, CA 93106-9660 USA

**Keywords:** Selective attention, Priority, Cognitive control, Exogenous attention, Endogenous attention, Spatial cueing, Working memory, Encoding, Maintenance

## Abstract

Physically salient stimuli are potent influences on behavior, but their negative impacts can be reduced in the presence of explicit goal-related cues. Here, we investigated whether goal-related cues associated with value are capable of insulating information from task-irrelevant abrupt onsets during two stages of information processing. Abrupt onsets were shown either after attention-directing cues and before a target (Experiment 1) or after a target that is to be remembered for later report (Experiment 2). The cues indicated the value associated with upcoming target locations, and they were either different in value, indicating that one was more valuable than the other, or equal in value. In both experiments, subjects were instructed to report the target that would earn them the most points (Experiment 1) or money (Experiment 2). In Experiment 1, performance suffered with equal cues, suggesting that orienting to multiple locations increases susceptibility to distraction from physically salient stimuli. In Experiment 2, the same pattern did not appear for abrupt onsets during the retention period; instead, the impact of the physically salient stimulus was dependent upon working memory capacity. The differential impact of abrupt onsets prior to (Experiment 1) and after (Experiment 2) encoding of value-related target locations suggest that physically salient task-irrelevant stimuli influence value-related information processing differently during orienting and maintenance.

## Introduction

Selective processing requires efficiently sampling information from the environment and maintaining the resulting internal representation to serve as the basis for decisions, actions, and memories. Selectivity is impacted by both the physical properties of a stimulus and internal influences related to our current goals (Carrasco, 2011; Corbetta & Shulman, 2002; Theeuwes, 2019). The balance between stimulus- and goal-driven influences depends on cognitive control, which is a function that can flexibly modulate selective information processing at multiple stages (Awh et al., 2006; Luck et al., 2021).

Physically salient abrupt onsets are important external influences that guide selective attention by alerting the system to changes in the environment (Breitmeyer & Ganz, 1976; Egeth & Yantis, 1997; Jonides & Yantis, 1988; Theeuwes, 1994; Yantis & Hillstrom, 1994). Early evidence indicated that abrupt onsets automatically capture attention (Jonides & Yantis, 1988; Remington et al., 1992; Yantis & Jonides, 1984), but subsequent studies demonstrate that the ability of abrupt onsets to involuntarily capture attention is tightly linked to attentional control settings (Folk et al., 1992; Ruthruff & Gaspelin, 2018; Yantis & Jonides, 1990). For instance, while search for abrupt onset targets is insensitive to set size, task-irrelevant abrupt onsets are not able to completely defy volitional goals, such as when spatial cues accurately inform subjects of the upcoming location of a target (Yantis & Jonides, 1990). Similarly, when abrupt onsets have different features from a target (Folk et al., 1992), they are able to be ignored. In other words, while abrupt onsets are able to involuntarily capture attention, this capture effect can be attenuated by goal-driven attention.

Goals are important internal influences that can guide the system using knowledge, expectations, and motivation. Multiple studies have demonstrated faster reaction times as well as more accurate responses with the prospect of reward (Bijleveld et al., 2010; Klink et al., 2017), a finding shown to correlate with neural activity related to proactive control following reward-associated cues (Frömer et al., 2021; Schevernels et al., 2014). While motivation provides a clear benefit for encoding, there are conflicting findings about the impact of motivation and value on prioritizing task-relevant information held in working memory. On the one hand, there is evidence for an increase in recall precision with monetary incentive; however, this only holds true when cues are presented prior to encoding as opposed to being presented retroactively (Brissenden et al., 2023). This finding suggests that reward enhances cognitive control by facilitating the proactive allocation of resources during encoding and not during maintenance. On the other hand, when items associated with a lower value are cued during maintenance, recall precision can increase to levels similar to recall precision for items associated with a higher value (Atkinson et al., 2022). This finding suggests that the motivational effects of reward on maintenance can be as powerful as explicit rehearsal cues. When considered together, it seems plausible that value-related motivation could increase control during various junctures along the information processing hierarchy.

Given that value and reward influence performance during both encoding and maintenance, it is reasonable to predict that task-relevant value may shield information from even the most potent distractors. We tested this prediction in two experiments by investigating whether value-associated information insulates information processing from distractors presented either after attention-directing cues, prior to encoding of the targets (Experiment 1), or during maintenance of task-relevant targets (Experiment 2). The results of Experiment 1 suggest that task-irrelevant abrupt onsets are most disruptive when 1) multiple spatial locations are associated with equal value and 2) presented latest in the cue–target interval. The results of Experiment 2 suggest improved performance when the task-irrelevant abrupt onset coincides with the location of valuable information, specifically for those with low working memory capacity.

## Experiment 1

**Fig. 1:**
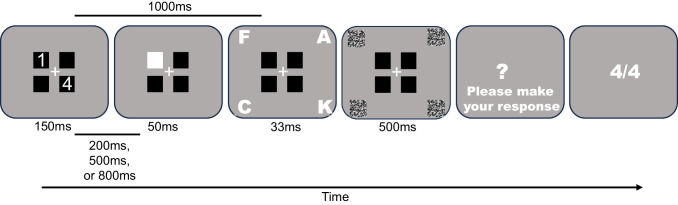
Sample trial sequence in Experiment 1. An example of a “different” cue trial with the abrupt onset occurring on the “low-value” cue location. The cue–target interval is 1,000 ms prior to the letter display and the abrupt onset could occur either 200, 500, or 800 ms into this interval. More specifically, if the abrupt onset occurred at 200 ms, there was a 750 ms blank period between the abrupt onset and the target display; if the abrupt onset occurred at 500 ms, there was a 450 ms blank period; and if the abrupt onset occurred at 800 ms, the there was a 150 ms blank period before the target

The goal of Experiment 1 was to investigate the impact of task-irrelevant abrupt onsets presented during a cue–target interval on the processing of value-related information. In the present study, subjects were presented with two simultaneous value-related cues parafoveally, indicating which subsequent targets to prioritize in the corresponding peripheral locations (Fig. 1). The value cues were either different or equal in value, requiring the subjects to choose which locations to attend to accordingly. An abrupt onset appeared during the cue–target interval on one of the parafoveal cue locations. Given the benefits of expected reward on allocating attention to value-associated information and promoting enhanced stimulus processing during encoding (Brissenden et al., 2023; Frömer et al., 2021; Krebs et al., 2013), subjects should appropriately allocate attention to the periphery in anticipation of the upcoming target display. When the cues are of different value, there may be no effect of the abrupt onset if attentional resources are predominantly allocated to the most valuable target location and when most of the display can be ignored. However, when the cues are of equal value, interference from abrupt onsets could be inescapable due to dividing attention across multiple target locations as well as the inability to filter out all but one target location in the display. When abrupt onsets appear in task-irrelevant locations, they can be immune from capture and properly ignored (Ruthruff & Gaspelin, 2018). However, it is unknown whether task-irrelevant abrupt onsets presented at potential cue locations influence value-driven orienting and encoding prior to the presentation of multiple targets in the periphery. Further, when the targets are of equal but low value, it is possible that the process of orienting attention to these targets is more susceptible to interference than when equal targets of high value are presented, as one might expect to be the case given that previous findings demonstrate a linear relationship between value and cognitive control (Frӧmer et al., 2021). It could also be the case that there is no difference between targets of equal low and equal high value, a finding that would be supported by research indicating that only differential values have an impact on attentional weights (Philiastides et al., 2010).

### Methods

#### Participants

Thirty-five adult students, 18–24 years of age (*M* = 18.97 ± 1.19; 11 reported men, 22 reported women) from the University of California, Santa Barbara (UCSB), volunteered to participate in the current study. Participants received one credit/hour (1.5 total credits) for their participation. All procedures were approved by the UCSB Human Subjects Committee.

#### Materials

All stimuli were generated using MATLAB with custom scripts and extensions from PsychToolbox and were presented on a CRT monitor with a refresh rate of 60 Hz and running on Linux. Subjects were seated approximately 94 cm away from the computer screen and used a standard keyboard to type their response.

#### Stimuli

Stimuli were presented on a gray background (RGB = 128, 128, 128). Four black squares (1.5° × 1.5°) were equally spaced around a white “ + ” presented at central fixation (0.5° × 0.5°). The number cues were white and presented in the center of their corresponding square (1.5°). The number cues were either equal in value (1 and 1; 4 and 4) or different in value (1 and 4; 4 and 1). The entire display size measured 16° × 12.8°. Each target letter (1.5°) was presented in a different peripheral corner of the display in white font. Target letters were masked by generating white Gaussian noise centered on gray (σ = 50) and matching the size of the target letters. The letters on each trial were drawn randomly without replacement from the alphabet (except for “m” and “z”).

#### Procedure

The task was a modified version of the multiple cue paradigm used to study shifts in endogenous attention (Oren et al., 2024). Each trial began with a center fixation jittered from 1,050 ms to 1,500 ms prior to the cue display. The two cue numbers were displayed for 150 ms followed by a 1,000 ms cue–target interval. The cues depicted the number of points associated with each corresponding peripheral target location. During the cue–target interval, the abrupt onset appeared for 50 ms at an onset of 200 ms, 500 ms, or 800 ms in the location of a high-value, low-value, or no-value associated parafoveal square (Note: “high” and “low” value locations are arbitrary in the equal cue condition). After the 1,000 ms cue–target interval, the four target letters appeared in the periphery for 33 ms before being immediately masked for 500 ms. Following the mask, subjects reported the target letter of their choice. Subjects were instructed to simply earn as many points as possible in the task. After their response, a feedback display appeared in the center of the screen for 2,000 ms, indicating how many points the subject earned out of the total possible number of points they could have earned on that given trial. Allowing the subject to choose which item to report is crucial to our design, as we are interested in the willful and voluntary allocation of attention. If we were to include a random response probe, we could only draw conclusions regarding that specific location or item, penalizing those that encoded the other equally rewarded location or item in the equal cue condition. The task consisted of 10 blocks with 36 trials each and was fully counterbalanced across all conditions of cue type, abrupt onset location, and abrupt onset time.

#### Analysis

The metric for performance in this experiment was the proportion of points earned relative to the maximum amount of possible points that could have been earned on a trial. Using the proportion of points earned as a proxy for performance ensures points are also given for suboptimal, though still correct, responses on different cue trials. Considering responses to the lower cue location in the different cue condition is important, as this could be an optimal strategy when the target in this location is the only one accurately remembered. This metric also ensures that 100% accuracy (proportion of points earned) reflects that the optimal choice was chosen regardless of condition. For example, choosing the optimal item to report in the “equal cue” condition would result in 1/1 = 1 or 4/4 = 1, and choosing the optimal choice in the “different cue” condition would result in 4/4 = 1. Two subjects were removed from analysis as outliers (performance was greater than 2.5 standard deviations from the mean).

### Results

**Fig. 2:**
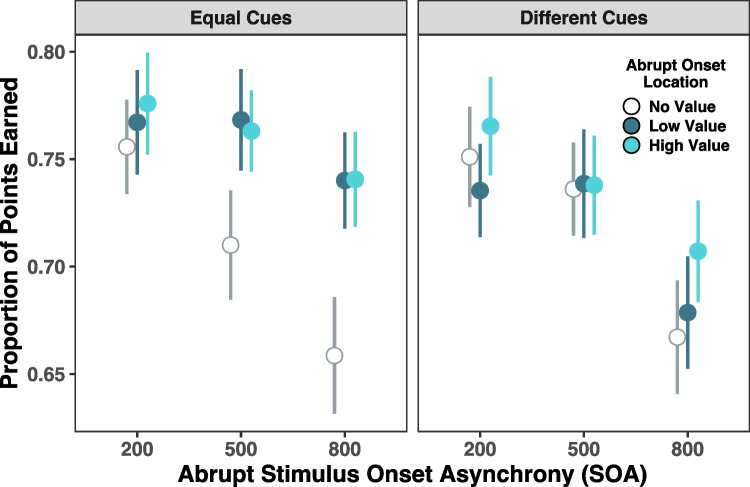
Results from Experiment 1. Figure displays means and standard errors of proportion of points earned

Mean points acquired as a function of cue type, abrupt onset location, and abrupt onset asynchrony are shown in Fig. 2. A repeated-measures analysis of variance (ANOVA) was conducted to assess the effects of cue type (equal, different), abrupt onset asynchrony (200 ms, 500 ms, or 800 ms), and abrupt onset location (no value, low value, high value) on the proportion of points earned. All post hoc comparisons were Bonferroni corrected. There was a significant main effect of cue type, *F*(1, 34) = 6.68, *p* = 0.014, η_p_^2^ = 0.164, with overall improved performance in the equal cue condition relative to the different value cue condition. There was also a main effect of abrupt onset asynchrony, *F*(2, 68) = 18.11, *p* < 0.001, η_p_^2^ = 0.348. Post hoc comparisons revealed that performance was significantly worse when the abrupt onset appeared late in the cue–target interval compared with when the abrupt onset appeared early (MD = 0.060, *SE* = 0.012), *t*(34) = 5.18, *p* < 0.001, *d* = 0.428, or in the middle of the cue–target interval (MD = 0.044, *SE* = 0.011), *t*(34) = 4.06, *p* < 0.001, *d* = 0.312. There was an additional main effect of abrupt onset location as well, *F*(2, 68) = 8.28, *p* < 0.001, η_p_^2^ = 0.196, with post hoc comparisons indicating significantly worse performance when the abrupt onset appeared on a no value cue location compared to when it appeared on either a low-value (MD = 0.025, *SE* = 0.009), *t*(34) = 2.64, *p* = 0.037, *d* = 0.178, or high-value (MD = 0.035, *SE* = 0.009), *t*(34) = 3.86, *p* = 0.001, *d* = 0.252, cue location.

A significant interaction between abrupt onset location and cue type also emerged, *F*(2, 68) = 5.85, *p* = 0.005, η_p_^2^ = 0.147. Post hoc comparisons indicated that the degradation in performance when the abrupt onset was presented on a no value cue location was driven by the equal cue condition as performance is significantly worse with presence of the abrupt onset on the no value cue location compared to the low (MD = 0.050, *SE* = 0.012), *t*(34) = 4.09, *p* = 0.004, *d* = 0.361, and high (MD = 0.052, *SE* = 0.011), *t*(34) = 4.70, *p* < 0.001, *d* = 0.371, value cue locations for the equal cue condition only (Fig. 2).

### Discussion

Experiment 1 investigated the competition between value-related information and task-irrelevant abrupt onsets during the preparatory period of priority control. Overall, we observed an impairment in performance as the abrupt onset appeared later in the cue–target interval, likely because after getting captured by the abrupt onset, there was insufficient time to reorient attention back to the cued location prior to the presentation of the target.

Perhaps more interesting is the pattern of results suggesting that there is only an effect of the location of the abrupt onset when cued target locations are of equal value. While the present study cannot delineate the mechanisms responsible for this pattern, it does suggest that equally prioritizing multiple locations promotes greater disruption from task-irrelevant abrupt onsets occurring in noninformative locations. We propose several potential explanations for these findings. If attention is divided across both equally valuable targets, the abrupt onset appearing in the parafoveal cue location could draw attention toward the center of the screen and away from the peripheral targets. If the abrupt onset is responsible for reorienting attention to the cue location, then performance should suffer the most when the abrupt onset occurs at a novel cue location for that trial, as this would delay the reorienting of attention to valuable locations in the periphery. This possible explanation is also consistent with the observation of degraded performance at longer abrupt onset SOAs. The present findings could also be explained by appealing to the quality of working memory representations. For example, holding multiple equally valuable items in memory may result in degraded representations of the locations, increasing their susceptibility to interference. This explanation is examined in Experiment 2.

## Experiment 2

The goal of Experiment 2 was to better understand the consequences of abrupt onsets for holding multiple value-related items in memory. If value-related cues enhance processing only at the encoding phase, abrupt onsets presented during retention should have little impact on the representations of targets presented in high-value locations. However, if the benefit of value-related cues manifests as altered maintenance, abrupt onsets should impact performance. Further, if the findings from Experiment 1 are consistent with an account based on degraded representations, the abrupt onset occurring in the place of a target associated with no value should continue to impair performance during retention in the equal cue condition.

To investigate this question, Experiment 2 was a modified version of Experiment 1, with several critical changes. First, the abrupt onset appeared in the retention interval after the target display and was placed on the location of one of the peripheral targets. Second, the value-related cues depicted monetary value (cents) as opposed to points, with the intention of increasing the motivational salience attached to the target locations. Finally, the targets were randomly oriented lines between 0° to 360°. For the response, subjects were required to first click on the location of the target that would earn them the most money and then make a continuous response matching the orientation of that target. Responses within 15° of the chosen target orientation were considered “correct” and resulted in the awarding of that target’s associated value. Using a continuous response measure affords the opportunity to assess whether the representations of equally valuable orientations are in fact weaker, resulting in more recall error, than representations that should be differentially prioritized. With the fidelity of these representations in question, it is important to consider individual working memory capacity as those with lower working memory capacity could lack the cognitive control necessary for maintaining multiple relevant representations in the face of distraction (Adam et al., 2015, 2018).

### Methods

#### Participants

Thirty-six adult students 18–24 years of age (*M* = 19.72 ± 1.45; 11 reported men, 24 reported women) from the University of California, Santa Barbara (UCSB), volunteered to participate in the current study. Participants received one credit/hour (1.5 total credits) for their participation. Additionally, participants were awarded money based on their task performance (up to $11.70). All procedures were approved by the UCSB Human Subjects Committee.

#### Materials

Stimuli for the change detection task were generated to directly replicate the change detection task created by Adam and colleagues (2018). All stimuli were created using MATLAB with custom scripts and extensions from PsychToolbox and were presented on a CRT monitor with a refresh rate of 85 Hz and running on Linux. Subjects were seated approximately 100 cm away from the computer screen and used a standard keyboard and mouse to record their response.

#### Stimuli

##### **Change detection task**

The set sizes used in this task were three, six, and eight. Ten unique colors were used for the square stimuli (RGB = 0, 0, 255; 0, 255, 0; 255, 0, 0; 255, 255, 0; 255, 0, 255; 0, 255, 255; 255, 128, 0; 255, 255, 255; 1, 1, 1) and the background was gray (RGB = 127.5, 127.5, 127.5). All stimuli were presented within a background display size of 7° × 5.2° with at least 1.8° of space between each item. The squares measured 1.2° × 1.2°, and the fixation measured 0.12° × 0.12°.

##### Main experiment

Stimuli were presented on a gray background (RGB = 128, 128, 128). Four black squares (1.5° × 1.5°) were equally spaced around a black oval presented at central fixation (0.4° × 0.4°). The number cues were white and presented in the center of their corresponding square (1.5°). The number cues were either equal in value (1 and 1; 4 and 4) or different in value (1 and 4; 4 and 1). The target display consisted of four black lines randomly oriented from 0° to 360° inside of four ovals outlined in black (1.5° × 1.5°) and presented in the peripheral corners of the display (16° × 12.8°). During the delay period, the abrupt onset occurred in either a high-value, low-value, or no-value target location in white for 50 ms. Responses were made with a computer mouse to report the target location associated with the most value. For the continuous response measure, the oval in the selected target location displayed a randomly oriented line that could be reoriented with the mouse until the subject pressed the space bar.

#### Procedure

##### Change detection task

Each trial began with a 1,000 ms fixation period followed by the presentation of either three, six, or eight different colored squares. The colored squares were displayed for 250 ms prior to a 1,000 ms delay period. One of the squares reappeared in either the same color (no change trial) or a different color (change trial) than the square previously presented in that location and remained on the screen until a response was made. Subjects were instructed to remain fixated on the center of the screen until it was time to make a response. For their response, subjects pressed the “s” key if the color was the *same* and pressed the “d” key if the color was *different*. There were 48 trials per set size and three blocks. Half of all the trials were a “change” trial and the other half a “no change” trial. On “change” trials, the square displayed in the response period was a different color than the square presented in that location during the encoding period. Capacity, as measured by *K*, was computed as follows: *N* × *(H − FA)*, where *N* represents set size, *H* refers to the hit rate, and *FA* refers to the false-alarm rate (Cowan, 2011). Each subject was then given an overall working memory capacity score, calculated as the average *K* across all set sizes. For the current study, we conducted a median split on all subject *K* scores (*Mdn* = 3.16*, Min* = 1.78*, Max* = 4.89*)*. All subjects with an average *K* score less than or equal to the median were considered “low capacity” individuals (*n* = 18) and all subjects with a *K* score above the median were considered “high capacity” individuals (*n* = 18*)*.

##### **Main experiment**

**Fig. 3:**
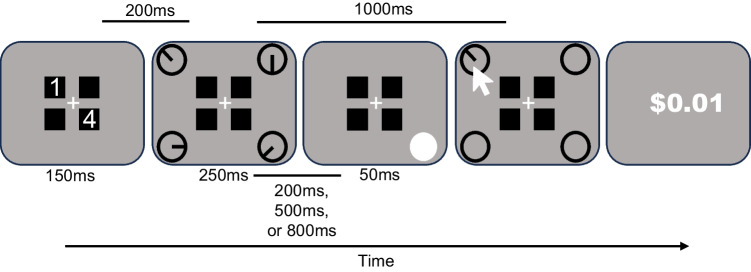
Sample trial sequence in Experiment 2. An example of a different cue trial with the abrupt onset occurring on the “high-value” orientation location. The retention interval is 1,000 ms following the orientation display and the abrupt onset could occur either 200 ms, 500 ms, or 800 ms into this interval. More specifically, if the abrupt onset occurred at 200 ms, there was a 750 ms blank period between the abrupt onset and the response display; if the abrupt onset occurred at 500 ms, there was a 450 ms blank period; and if the abrupt onset occurred at 800 ms, the there was a 150 ms blank period before the response display. The white arrow depicts the subject’s cursor when making a response

Each trial began with a center fixation jittered from 1,050 ms to 1,500 ms prior to the cue display. The two cue numbers were displayed for 150 ms followed by a 200 ms interstimulus interval prior to the target display. The number cues depicted the amount of money (in cents) associated with each peripheral location. The target display was presented for 250 ms and followed by a 1,000 ms retention interval. During the retention interval, an abrupt onset, a white filled oval, occurred in the location of a target associated with either a high value, low value, or no value for 50 ms. Importantly, the abrupt onset occurred either 200 ms, 500 ms, or 800 ms into the retention interval. Following the retention period, outlined ovals were presented in all previously displayed target locations until the subject chose the location of the target that they believed would earn them the most money. After indicating the appropriate target location, the oval displayed a randomly oriented line for the subject to adjust accordingly until the space bar was pressed marking the end of the response period. If the subject reported an orientation within 15° of the actual orientation in the chosen target location, they received the money associated with that target. After making their response, subjects were shown feedback for 2,000 ms informing them of how much money they earned for that trial. The task consisted of 10 blocks with 36 trials each and was fully counterbalanced across all conditions of cue type, abrupt onset location, and abrupt onset time Fig. 3.

### Results

#### Performance as proportion of money earned

**Fig. 4:**
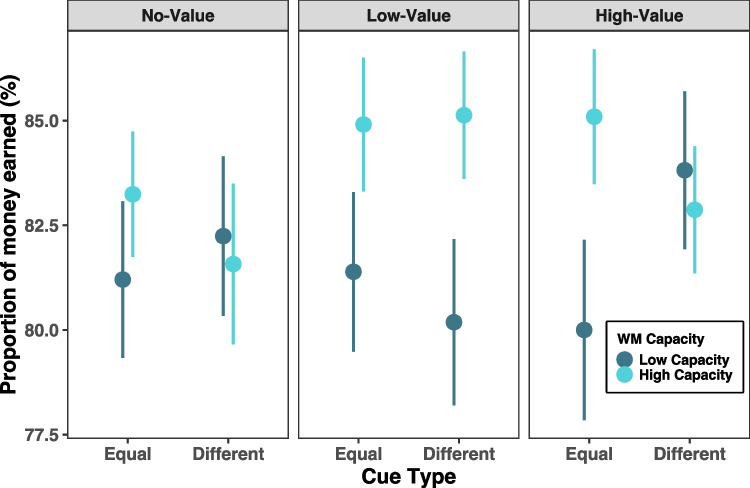
Results from Experiment 2 for proportion of money earned. Figure displays means and standard errors for proportion of money earned out of the total amount of money that could have been earned

To directly compare the results from Experiment 2 with Experiment 1, performance was first quantified as the proportion of money earned out of the total amount of money that could be earned on a trial. A mixed-design ANOVA was conducted to assess the effects of cue type (equal, different), abrupt onset asynchrony (200 ms, 500 ms, or 800 ms), abrupt onset location (no value, low value, high value), and the between-subjects factor of working memory capacity (high, low) on the proportion of money earned. All post hoc comparisons were Bonferroni corrected. Unlike Experiment 1, there were no main effects of cue type, abrupt onset asynchrony, or abrupt onset location. However, there was a three-way interaction between working memory capacity, cue type, and abrupt onset location, *F*(2, 68) = 4.09, *p* = 0.021, η_p_^2^ = 0.107. To understand this interaction, three separate mixed-design ANOVAs were conducted to investigate how the simple interaction of cue type and working memory capacity differs across abrupt onset locations. A significant two-way interaction between cue type and capacity emerged for the high-value condition only, *F*(1, 34) = 10.14, *p* = 0.003, η_p_^2^ = 0.230. Follow-up post hoc comparisons show that when the abrupt onset occurs in the location of a high-value target, performance is significantly better in the different cue compared to equal cue condition for those with low working memory capacity (MD = 0.038, *SE* = 0.013), *t*(34) = 2.85, *p* = 0.045, *d* = 0.284 (Fig. 4).

These results clearly show a different pattern than Experiment 1, as no main effects proved to be significant. However, the cue type and location of the abrupt onset did matter when considering working memory capacity. When one target was more valuable than the other, performance significantly improved when the abrupt onset appeared in the location of the high-value target, but only for those with low memory capacity.

#### Absolute recall error

**Fig. 5:**
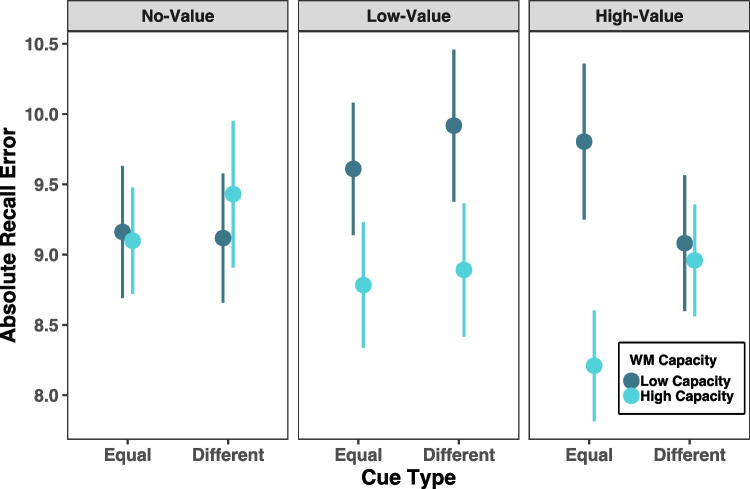
Results from Experiment 2 for absolute response distance. Figure displays means and standard errors of the absolute value of the response error in degrees

Performance was also quantified in this task as recall error, or the absolute angular distance between the reported angle and the true angle at the location the subject chose with their cursor. A mixed-design ANOVA was again run with the same variables of cue type (equal, different), abrupt onset asynchrony (200 ms, 500 ms, 800 ms), abrupt onset location (no value, low value, or high value), and working memory capacity (low, high) on absolute recall error, with working memory capacity, *K*, as the only between-subjects factor. A three-way interaction between capacity, cue type, and abrupt onset location also emerged, *F*(2, 68) = 3.53, *p* = 0.035, η_p_^2^ = 0.094. To break down the three-way interaction, we submitted each level of abrupt onset location to separate mixed-design ANOVAs. There was a significant two-way interaction between capacity and cue type when the abrupt onset occurred in the high-value location only, *F*(1, 34) = 9.01, *p* = 0.005, η_p_^2^ = 0.209. However, this finding should be interpreted with caution as no significant differences survived post hoc comparisons (Fig. 5).

### Discussion

The purpose of Experiment 2 was to understand how value-related cues and task-irrelevant abrupt onsets collectively modulate the retention of value-related items in working memory. Experiment 2 was also necessary to further elucidate the findings from Experiment 1, which suggested that degraded representations in the equal cue condition could potentially explain the impaired performance observed when the abrupt onset occurred in an uninformative location during the cue–target interval. We observed that for those with low working memory capacity, performance was dependent upon the location of the abrupt onset and whether one or two targets were to be prioritized. Low working memory capacity individuals performed better when an abrupt onset was presented in place of a high-value target in the different cue condition compared to when the abrupt onset occurred on any target in the equal cue condition. For individuals with low working memory capacity, an abrupt onset presented at a highly valuable target location may facilitate performance by acting like a rehearsal cue. It is possible this pattern would not be seen in high-capacity individuals as their performance was already very high, leaving little room for improvement. Relatedly, previous work has found that high-capacity individuals suffer from larger performance costs when probes are placed in target locations, perhaps due to the added disruption a probe mask elicits when attention is already very tightly focused (Fukuda & Vogel, 2009). In the absence of the abrupt onset acting as a rehearsal cue in the equal cue condition, those with low working memory capacity may be more susceptible to interference due to the difficulty in accurately holding multiple representations at once. Another possible explanation for this pattern is that subjects chose to remember only one of the equally valuable targets and in turn were subjected to interference from the abrupt onset when it occurred in the place of the other equally valuable target.

## General discussion

The present series of experiments presented an abrupt onset during two distinct phases of priority control (cue–target preparation and maintenance) while actively prioritizing task-relevant value-related information. In both experiments, two value-related cues were presented near the center of the screen indicating which peripheral target locations to prioritize. Critically, the valuable cues could either be equal or different, requiring the subject to decide which locations to prioritize on any given trial. The abrupt onset occurred on a location associated with no value, a low value, or a high value. In the equal cue condition, a low-value location would be either location when the equal cues were both 1’s, and a high value location would be either location when the equal cues were both 4’s. In Experiment 1, the abrupt onset appeared in the cue location and was presented either early, in the middle of, or late into the cue–target interval. In Experiment 2, the abrupt onset appeared in the location of the target and was presented either early, in the middle of, or late into the retention period.

The purpose of Experiment 1 was to investigate how abrupt onsets interact with goal-directed orienting to valuable information when presented after a spatial cue, but prior to encoding of the target. We found that the timing of the abrupt onset mattered, with performance decreasing as a function of when the abrupt onset occurred in the cue–target interval. We propose that performance may have suffered when the abrupt onset appeared shortly before the target display due to the limited amount of time remaining in the cue–target interval to reorient attention back to the appropriate target location (Cashdollar et al., 2013). Unique to the equal cue condition, performance was impaired when the abrupt onset appeared in a location that did not depict any value at all. There are two potential explanations for this finding. First, it is possible that in the equal cue condition, subjects are dividing their attention across both valuable target locations. If this division of attention amounts to diffusely attending to a larger contiguous region of space (Herrmann et al., 2010), this attended region may also include the parafoveal cue locations, resulting in an increased likelihood of interference on the presentation of the abrupt onset (Belopolsky et al., 2007). Second, it is also possible that remembering multiple target locations involves a division of resources that results in two less precise working memory representations compared with one more stable representation (Sprague et al., 2014). If the findings of Experiment 1 were driven by degraded memory representations in the equal cue condition, then there should be a similar decline in performance in the equal cue condition when the abrupt onset coincides with the location of a target worth no value during the retention period in Experiment 2. However, this was not the case, as performance was similar for equal and different cue conditions when the abrupt onset appeared in an uninformative location, suggesting that the pattern of evidence is not consistent with the degradation of spatial memory representations.

The goal of Experiment 2 was to investigate the influence of abrupt onsets on value-related information already encoded into working memory. Interestingly, the effects of cue type, abrupt onset location, and abrupt onset timing observed in Experiment 1 did not extend to Experiment 2. Instead, when the abrupt onset occurred during the retention period, its location and time of onset had no influence on performance. However, when subjects’ working memory capacity is considered, abrupt onsets presented in the location of a high-value target improved performance for those with low working memory capacity. In effect, it appears as though the abrupt onsets may provide a “cue” for low-capacity individuals. In line with previous work, the abrupt onset could facilitate performance by inadvertently refreshing attention to the highly valuable target location (Atkinson et al., 2022). Previous research has demonstrated that spatial attention during maintenance contributes to the improved recall of information at that location, highlighting the importance of spatial rehearsal during retention (Awh et al., 1998). These results are also consistent with evidence suggesting that when instructional cues are used to elicit spatial rehearsal, less valuable items are recalled with the same level of accuracy as highly valuable items (Atkinson et al., 2022). It is plausible that those with low working memory capacity lack the attentional control necessary to rehearse prioritized locations, which could explain why the abrupt onset only facilitated performance for these individuals (Adam et al., 2015, 2018). From previous work, we know that individuals with low working memory capacity have difficulty filtering out irrelevant information (Vogel et al., 2005). Additionally, while low- and high-capacity individuals are similarly captured by salient distractors, low-capacity individuals have substantial difficulty suppressing these distractors following capture (Gaspar et al., 2016). When the abrupt onset is placed on a target location in the present study, failure to suppress this abrupt onset location could partially explain the enhanced performance in this condition for low-capacity individuals. Additionally, it has been shown that when several items are associated with graded priority, high-priority items are most susceptible to interference from distraction; however, when low-priority information can be discarded, this vulnerability is protected. This highlights an added potential explanation for increased performance in the different cue as opposed to equal cue condition for low-capacity individuals, with the abrupt onset in the high-priority location promoting the removal of low-priority information (Lorenc et al., 2021).

Overall, our results are consistent with the notion that abrupt onsets influence value-related information processing differentially prior to encoding and during retention. Although the performance impairment observed in Experiment 1 is in line with a divided attention account rather than a degraded spatial memory representation account, we did not measure individual differences related to working memory capacity and thus we cannot completely rule this out as a potential explanation. When incorporating working memory capacity in Experiment 2, we observed a surprising finding that not only provides evidence for previous accounts of priority control during maintenance (Atkinson et al., 2022) but may also help explain how these strategies differ amongst individuals. Given that those with low working memory capacity appear to benefit from spatial rehearsal, there could be deficits in these individuals related to cognitive control that are also associated with the allocation of attention (Bleckley et al., 2003; Kane & Engle, 2003; Vogel et al., 2005). It is important to highlight the unique task designs and reward structures between our two experiments and their potential to limit the generalizability of our findings. Changing the reward earned from points to monetary value in Experiment 2 was done simply to enhance the size of the experimental effect via increasing the motivational effect of reward and subsequent motivation to prioritize value-related information. It is unlikely this modification would fundamentally change our results as previous research has shown that both points and monetary value are capable of enhancing attentional control (Shen & Chun, 2011; Walsh et al., 2021). Therefore, we believe monetary value may have only strengthened the priority structure in Experiment 2 (Hübner & Schlösser, 2010). Additionally, the memoranda were changed from letters in Experiment 1 to orientations in Experiment 2. Using orientations allowed for a continuous measure of individual responses (i.e., recall error) as opposed to the binary outcome variable in Experiment 1 (i.e., correct or incorrect). While we recognize that changing these two factors introduces a potential confound, we felt that the potential gain from enhancing the value-driven effect with reward and using a continuous response was necessary. Future research should consider utilizing the same exact stimuli in both experiments to more tightly control for the influence of abrupt onsets during preparatory encoding and maintenance of the same type of valuable information.

## Concluding remarks

Previous work indicates that there is diminished capture by abrupt onsets in the presence of explicit goals. Here, we investigated whether motivational salience related to value can protect one from the distracting effects of abrupt onsets. We found that task-irrelevant abrupt onsets influence value-related information processing differently during preparatory encoding and maintenance. Abrupt onsets were most disruptive when presented just prior to the target display and when attention was allocated to multiple valuable target locations. We also found that when one item was more valuable than another, abrupt onsets presented in the more valuable location increased performance for those with low working memory capacity. These findings not only contribute to our current understanding of stimulus- and goal-driven attention but also provide clarification as to how these two distinct properties of attention interact during two critical stages of information processing.

## Data Availability

De-identified data available upon request.

## Code availability

All custom scripts are available upon request.
